# Gender (in) differences in prevalence and incidence of traumatic experiences among orphaned and separated children living in five low- and middle-income countries

**DOI:** 10.1017/gmh.2015.1

**Published:** 2015-04-24

**Authors:** C. L. Gray, B. W. Pence, J. Ostermann, R. A. Whetten, K. O'Donnell, N. M. Thielman, K. Whetten

**Affiliations:** 1 Department of Epidemiology, Gillings School of Global Public Health, University of North Carolina at Chapel Hill, CB #7435, Chapel Hill, North Carolina, USA; 2 Center for Health Policy and Inequalities Research, Duke Global Health Institute, Duke University, Box 90519, Durham, North Carolina, USA; 3 Departments of Psychiatry and Pediatrics, DUMC #3364, Duke University Medical Center, Durham, North Carolina, USA; 4 Center for Child and Family Health, Duke University, 411 West Chapel Hill Street, Suite 908, Durham, North Carolina, USA; 5 Department of Medicine, DUMC #3152, Division of Infectious Diseases and International Health, Duke University, Durham, North Carolina, USA; 6 Sanford school of Public Policy, Duke University, Box 90239, Durham, North Carolina, USA

**Keywords:** gender, orphans, low- and middle-income countries (LMIC), potentially traumatic events, prevalence, incidence

## Abstract

**Background.:**

Approximately 153 million children worldwide are orphaned and vulnerable to potentially traumatic events (PTEs). Gender differences in PTEs in low- and middle-income countries (LMIC) are not well-understood, although support services and prevention programs often primarily involve girls.

**Methods.:**

The Positive Outcomes for Orphans study used a two-stage, cluster-randomized sampling design to identify 2837 orphaned and separated children (OSC) in five LMIC in sub-Saharan Africa and Asia. We examined self-reported prevalence and incidence of several PTE types, including physical and sexual abuse, among 2235 children who were ≥10 years at baseline or follow-up, with a focus on gender comparisons.

**Results.:**

Lifetime prevalence by age 13 of any PTE other than loss of a parent was similar in both boys [91.7% (95% confidence interval (CI) (85.0–95.5)] and girls [90.3% CI (84.2–94.1)] in institutional-based care, and boys [92.0% (CI 89.0–94.2)] and girls [92.9% CI (89.8–95.1)] in family-based care; annual incidence was similarly comparable between institution dwelling boys [23.6% CI (19.1,−29.3)] and girls [23.6% CI (18.6,−30.0)], as well as between family-dwelling boys [30.7% CI (28.0,−33.6)] and girls [29.3% CI (26.8,−32.0)]. Physical and sexual abuse had the highest overall annual incidence of any trauma type for institution-based OSC [12.9% CI (9.6–17.4)] and family-based OSC [19.4% CI (14.5–26.1)], although estimates in each setting were no different between genders.

**Conclusion.:**

Prevalence and annual incidence of PTEs were high among OSC in general, but gender-specific estimates were comparable. Although support services and prevention programs are essential for female OSC, programs for male OSC are equally important.

## Introduction

As the world's 153 million orphans and still more separated children (UNICEF, 2012) continue to grow in number, leaders and policymakers face an ongoing challenge to provide them an environment that supports positive growth and development into adulthood. Lower- and middle-income countries (LMIC) have disproportionately higher rates of orphaning, in part due to HIV/AIDS; 17 million of the world's orphans are AIDS orphans (UNICEF, 2012).

Orphans and separated children (OSC) in LMIC are vulnerable to a number of additional potentially traumatic events (PTEs) beyond the loss of a parent, such as war, abuse, and being forced from their home or care setting (Whetten *et al*. 2011). In general, traumatic events are exposures to actual or threatened death, serious injury or sexual violation (American Psychiatric Association, 2013). PTEs are events of such gravity that they have the potential to be traumatic, but not all individuals have a traumatic response to the same PTE. PTEs have been associated with emotional difficulties and lower cognitive performance in OSC (Escueta *et al*. 2014). However, few studies have quantified the burden of PTEs in OSC. Li *et al.*  examined PTEs in Chinese children affected by AIDS, including but not focused on orphans; results indicated high burden of PTEs and associations of PTEs with depression, anxiety and posttraumatic stress (Li *et al.* 2009). In a study in Kenya, Atwoli *et al.*  assessed adolescents for PTEs, including bullying and physical and sexual abuse; PTEs in street youth in particular were associated with posttraumatic stress disorder (PTSD; Atwoli *et al.* 2014).

Several published studies have examined medical care and support services (Chaudhry *et al.* 1995; Ekabua *et al.* 2006; Ige & Fawole, 2012), tested integration of counseling services (Kim *et al.* 2009), and described prevention programs for children in LMIC who have experienced PTEs (Sarnquist *et al.* 2014; Sommarin *et al.* 2014). These studies have focused on girls; interventions or support services with a focus on, or even an inclusion of, boys are less common. Characterization of child assault based on use of clinical services suggests occurrence is almost exclusively in girls (Birdthistle *et al.* 2011). Yet, the few previous community-based studies of the occurrence of PTEs in orphans or OSC report that boys and girls are at comparable risk of PTEs (Li *et al.* 2009; Whetten *et al*. 2011; Atwoli *et al.* 2014).

Understanding the relationship between gender and both prevalence and incidence of PTEs among OSC in LMIC can provide useful information for leaders and policymakers seeking to establish or expand support services or prevention programs. The Positive Outcomes for Orphans (POFO) study is a longitudinal cohort that provides a unique opportunity to examine both prevalent and incident PTEs in five diverse LMIC in a representative sample of OSC. We quantified the burden of PTEs in OSC, including physical and sexual abuse. Specifically, we focused on comparisons by gender to better understand which children are at risk for PTEs in general, and for abuse in particular, to inform interventions aimed at reducing the risk and sequelae of PTEs in the OSC population.

## Methods

### Population

POFO is a longitudinal study of OSC followed for seven rounds of data collection over a 36 month period at six culturally, politically and geographically diverse sites in five LMIC: Battambang District, Cambodia; Addis Ababa, Ethiopia; Hyderabad, India; Nagaland, India; Bungoma District, Kenya; and Kilimanjaro Region, Tanzania.

Data collection began between May 2006 and February 2008, depending on study site. OSC ages 6–12 at baseline were randomly sampled in both institution-based and family-based settings through a two-stage design to identify a statistically representative sample of the OSC population in the regions in which the study was conducted; 2837 OSC participated in the POFO study.

Details of the sampling frame have been described elsewhere (Whetten *et al*. 2009, 2011). Briefly, 250 institution-dwelling OSC and 250 family-dwelling OSC were targeted from each study site. Institutions, defined as structures with at least five OSC from at least two different families, were randomly sampled from a comprehensive list of all institutions in each study site. Up to 20 children were randomly sampled from each institution. Family-dwelling OSC were sampled using 50 geographic clusters defined at each study site. Up to five children from each cluster were randomly sampled using lists or a house-to-house census.

### Measures

Interviews were conducted at baseline and at approximately 6-month intervals for up to 3 years. Questions about potentially traumatic experiences were asked at baseline and then every other round of data collection (approximately annually) for a total of four interviews.

#### Child characteristics

Demographic information collected at baseline included gender, age, setting (institution-dwelling or family-dwelling), and OSC type (single orphan, double orphan, separated, neither; maternal orphan, paternal orphan or both).

#### Potentially traumatic events

Potentially traumatic experiences were assessed using the Life Events Checklist (LEC), a tool developed by the National Center for Posttraumatic Stress Disorder to aid in the detection of PTSD (Gray *et al.* 2004). The LEC has been validated in multiple populations and is widely used across different cultures (Gray *et al.* 2004; Elhai *et al.* 2005). Children in the POFO study responded to a 17-item list of ‘things I have seen and heard’; they could indicate whether the event had been experienced one time, more than one time or not at all. At the first interview, the child indicated whether he or she had ever experienced the event. At subsequent interviews, the child reported if he or she had experienced the event in the past year (i.e., approximately since the last trauma assessment), prior to the past year or both.

We used self-reported trauma because a prior study in the POFO population showed that caregivers underreported PTEs experienced by OSC (Rajan *et al*. 2013). Trauma measures were not administered to children <10 years of age because pilot testing indicated that younger children could not be expected to reliably understand and answer such questions, and the local Institutional Review Boards (IRB) did not permit asking children under 10 about traumatic events. Therefore, our analyses included potentially traumatic experiences self-reported by OSC ages ≥10 at the time of a given assessment. Some OSC who were too young to report PTEs at baseline were included in subsequent rounds.

As was previously done to describe traumatic experiences in this population (Whetten *et al*. 2011) and similar to other work (Mugavero *et al*. 2006), we collapsed the 17 PTEs into six mutually exclusive categories: disasters or accidents (Cronbach's alpha = 0.35); war, riots or killings (alpha = 0.56); physical or sexual abuse (alpha = 0.40); witnessing violence in the care setting (alpha = 0.26); witnessing family death (alpha = 0.23); and being forced to leave the home or care setting (alpha = 0.27). For a complete list of PTEs in each category, please see Appendix Table A1. A category was considered endorsed if any of its constituent items was endorsed. Death of a parent or separation from parents was the defining characteristic for inclusion in the study and was not included as a PTE unless the child personally witnessed a parental death. In that case, the event was captured in the category ‘witnessing family death’.

We also created a variable representing ‘any’ trauma. Endorsement of any of the six trauma categories was used to classify the broader experience of any trauma (alpha = 0.65).

We defined lifetime prevalence as a report of ever experiencing the event by the time of a given interview. At follow-up surveys, we defined incident trauma as a report of experiencing the event in the past year (approximately since the last trauma measurement). An event was considered incident regardless of whether that event had been experienced previously.

#### Anxiety

We used the PTSD Checklist – Specific (PCL-S), which has been validated in multiple populations, to assess anxiety (Weathers *et al*. 1991, 1993; Blanchard *et al*. 1996). The PCL-S is a 17-item checklist in which each item is endorsed in terms of severity on a 5-point scale. While the PCL-S can be used to classify individuals who likely meet criteria for PTSD, we used a continuous measure of the score (range 17–85) and report the outcome as ‘anxiety’ (Cronbach's alpha = 0.95).

### Analyses

The analysis sample was restricted to interviews at which a child's current age was at least 10 years.

Lifetime prevalence by age 13 was estimated using logistic regression. Age 13 was selected because it represents a natural cut-point in child development (puberty) and because it was an age that the majority of participants experienced over the course of the longitudinal study. To calculate lifetime prevalence at age 13 in the final round of follow-up, we fit a model including age, a squared term for age and study round. Additionally, we summed the number of types of events experienced for each trauma category and fit a linear regression model with the same age and round covariates to estimate the mean number of items endorsed in each trauma category.

Annual (12 month) incidence was estimated as the proportion and 95% confidence interval (CI) of children reporting each type of PTE across all completed follow-up surveys. We also calculated the mean number of types of incident events endorsed for each category.

Analyses were stratified by setting (institution-based care and family-based care) to reflect the sampling structure of the two OSC groups. Analyses accounted for the complex survey design by incorporating stratification by site, clustering by sampling unit and sampling weights to account for varying probabilities of selection. Design and specification of the site and sampling unit levels has been described elsewhere (Whetten *et al*. 2009).

We used Kaplan–Meier survival methods and log-rank tests to compare gender-stratified estimates of the age at which any reported trauma, and physical and sexual abuse specifically, first occurred. Since children did not start reporting traumatic experiences until age 10, the first step of the survival function reflects the probability of trauma-free survival at age 10, estimated as the proportion of children entering follow-up at age 10 who reported no lifetime trauma. Children reporting no lifetime history of trauma at entry then contributed person-time at risk until the report of an incident experience (representing an event) or end of follow-up (censoring). Children who were >10 years old at entry (e.g. enrolled at age 11 or 12) and who reported a lifetime experience of trauma were excluded from the survival analysis altogether since the timing of the occurrence of the prevalent event (before or after age 10) could not be determined. Estimates of abuse-free survival proceeded similarly.

We estimated the association of lifetime exposure to PTEs with current anxiety symptoms using a linear mixed model estimated on all available interviews (i.e. multiple observations per participant). We used gender-stratified models which included fixed effects for sites and random intercepts for sampling units (institutions or community geographic clusters) within sites and participants within sampling units. The models were further adjusted for age, setting and study site.

To assess how selection bias due to missed interviews in our longitudinal dataset may have impacted our results, we combined inverse probability of observation weights with our sampling weights (Seaman & White, 2013). We assumed that data were missing at random, conditional on observed covariates and used logistic regression to calculate the predicted probability of a child being observed at a given round, conditional on gender, setting (institution *v.* family-based care), current age, site and study round. We multiplied the inverse of this probability (an inverse probability of observation weight) by the population sampling weight and re-ran our analyses.

All analyses were conducted using Stata 13 (StataCorp, 2013).

The POFO study was approved by the IRB at Duke University and at each of the study sites. Caregiver consent and child assent was obtained and recorded on IRB-approved consent forms. Interviewers were trained on site-specific protocols for addressing timely information such as reports of recent abuse, including working with local agencies to ensure protection of the children.

## Results

### Sample characteristics

In total, 2235 OSC (1053 institution-dwelling and 1182 family-dwelling) were at least 10 years of age at one or more interviews and were included in this analysis (Table 1). Over half were male among both institution-dwelling OSC (58%) as well as family-dwelling OSC (53%). Most institution-dwelling (74%) and most family-dwelling OSC (78%) were either 10 or 11 years old at their first trauma-related interview. Only 10% of family-dwelling OSC were double orphans, whereas 39% of institution-dwelling OSC were double orphans. In both settings, single orphans were much more likely to have lost a father than a mother: 34% *v.* 10% in institution-dwelling OSC and 56% *v.* 16% in family-dwelling OSC.

**Table 1.** Characteristics of institution-based and family-based OSC

Characteristic (*N* = 2235)	Institution-based OSC (*N* = 1053)		Family-based OSC (*N* = 1182)	
	*N*	(%)	*N*	(%)
*Gender*				
Male	614	58.3	631	53.4
Female	439	41.7	551	46.6
*Site*				
Cambodia	112	10.6	199	16.8
Ethiopia	175	16.6	192	16.2
Hyderabad	209	19.8	222	18.8
Kenya	188	17.9	192	16.2
Nagaland	150	14.2	163	13.8
Tanzania	219	20.8	214	18.1
*Age at first trauma interview*				
10	395	37.5	457	38.7
11	379	36.0	462	39.1
12	214	20.3	210	17.8
13	56	5.3	45	3.8
14	4	0.4	7	0.6
15+	5	0.5	1	0.1
*OSC deceased parent*				
Neither (separated)	178	16.9	120	10.2
Mother	105	10.0	188	15.9
Father	358	34.0	663	56.1
Both	412	39.1	211	10.2

### Prevalence of trauma

Over 90% of OSC in both institution-based and family-based settings had experienced at least one PTE by age 13, not including the loss of a parent (Table 2). Boys and girls had comparable predicted prevalence in both institution-based [91.7% CI (85.0–95.5) *v.* 90.3% CI (84.2–94.1), respectively] and family-based settings [92.0% CI (89.0–94.2) *v*. 92.9% CI (89.8–95.1), respectively]. Certain types of traumas were particularly prevalent. Witnessing a family death (which could be but was not necessarily the death of a parent) was the most commonly reported category among both institution-dwelling OSC [72.5% CI (67.7–76.9)] and family-dwelling OSC [71.8% CI (68.5–74.9)]. Predicted prevalence of physical and sexual abuses was also high, estimated at 50.3% CI (42.5–58.0) among institution-dwelling OSC and 54.0% CI (50.2–57.7) among family-dwelling OSC.

**Table 2.** Predicted trauma prevalence at age 13 among institution-based and family-based OSC, overall and gender-stratified

	Institution-based OSC						Family-based OSC					
	Overall		Male		Female		Overall		Male		Female	
Trauma type^a^	Mean no. items^b^	% (95% CI)	Mean no. items	% (95% CI)	Mean no. items	% (95% CI)	Mean no. items	% (95% CI)	Mean no. items	% (95% CI)	Mean no. items	% (95% CI)
Disaster or accidents (range: 0–3)	0.09	5.9 (3.7–9.5)	0.09	6.7 (3.9–11.4)	0.08	4.8 (2.6–8.7)	0.12	9.5 (7.5–11.9)	0.12	8.8 (6.4–11.9)	0.13	10.3 (7.5–14.1)
War, riots, killings (range: 0–3)	0.34	20.0 (14.5–26.8)	0.34	19.8 (13.9–27.4)	0.34	20.0 (13.3–29.1)	0.32	22.0 (19.2–25.0)	0.33	22.6 (19.1–26.7)	0.31	21.2 (17.0–26.1)
Forced to leave home or care setting (range: 0–2)	0.32	27.6 (21.2–35.2)	0.33	28.2 (20.6–37.3)	0.31	26.7 (19.0–36.2)	0.16	15.0 (12.5–17.9)	0.15	13.6 (10.6–17.2)	0.18	16.6 (13.4–20.5)
Violence in family or care setting (range: 0–3)	0.37	30.9 (25.1–37.4)	0.37	31.1 (23.8–39.4)	0.38	30.9 (22.6–40.6)	0.43	36.8 (33.1–40.7)	0.43	36.8 (32.0–41.9)	0.44	36.9 (31.9–42.2)
Physical or sexual abuse (range: 0–4)	0.85	50.3 (42.5–58.0)	0.88	49.4 (39.9–59.0)	0.80	51.3 (42.0–60.5)	0.88	54.0 (50.2–57.7)	0.91	53.9 (48.9–58.9)	0.84	54.0 (49.0–59.0)
Witnessing a family death (range: 0–2)	0.86	72.5 (67.7–76.9)	0.87	73.9 (68.8–78.5)	0.85	70.6 (62.4–77.7)	0.89	71.8 (68.5–74.9)	0.87	70.2 (65.5–74.6)	0.92	73.5 (69.1–77.5)
Any trauma (range: 0–17)	2.83	91.0 (85.6–94.5)	2.88	91.7 (85.0–95.5)	2.75	90.3 (84.2–94.1)	2.80	92.4 (90.3–94.0)	2.79	92.0 (89.0–94.2)	2.81	92.9 (89.8–95.1)
Repeated traumatic events (range: 0–17)^c^	1.10	53.3 (46.6–59.9)	1.11	52.6 (43.9–61.1)	1.07	54.3 (45.9–62.4)	1.27	57.2 (53.1–61.1)	1.30	56.6 (51.0–62.1)	1.25	57.8 (52.4–63.1)

a A category was considered endorsed if any of the items in it was endorsed. The total number of items for a category is indicated by the range.

b Mean number of items endorsed in the category.

c The event was endorsed as occurring ‘two or more’ times.

### Incidence of trauma

More than 20% of institution-dwelling OSC and 30% of family-dwelling OSC per year experienced an incident traumatic event. Annual incidence of PTEs among institution-dwelling boys [23.6% CI (19.1–29.3)] was the same as among girls [23.6% CI (18.6–30.0)]. Similarly, annual incidence among family-based boys [30.7% (CI 28.0–33.6)] was comparable with that among girls [29.3% CI (26.8–32.0)]. By far, abuse was the PTE with the highest annual incidence: 12.9% CI (9.6–17.4) of institution-dwelling OSC and 19.4% CI (14.5–26.1) family-dwelling OSC per year reported experiencing physical or sexual abuse during longitudinal follow-up.

### Gender comparisons

Comparing male and female OSC, both prevalence and annual incidence were nearly the same for each specific type of trauma, regardless of care setting (Tables 2 and 3). In particular, the predicted prevalence by age 13 of physical and sexual abuse was 49.4% CI (39.9–59.0) among institution-dwelling boys, compared with 51.3% CI (42.0–60.5) among girls; in family-based care, predicted prevalence by age 13 was 53.9% CI (48.9–58.9) among boys and 54.0% CI (49.0–59.0) among girls. Annual incidence of physical and sexual abuse was also similar between the two genders in each setting: 13.6% CI (9.9–18.5) among boys and 12.0% CI (18.5–17.1) among girls in institution-based care, and 19.8% CI (17.6–22.2) among boys and 19.0% CI (16.7–21.7) among girls in family-based care.

**Table 3.** Twelve-month incidence of traumatic events at age 13 among institution-based and family-based OSC, overall and gender-stratified

	Institution-based OSC						Family-based OSC					
	Overall		Male		Female		Overall		Male		Female	
Trauma type	Mean no. items	% (95% CI)	Mean no. items	% (95% CI)	Mean no. items	% (95% CI)	Mean no. items	% (95% CI)	Mean no. items	% (95% CI)	Mean no. items	% (95% CI)
Disaster or accidents (range: 0–3)	0.01	0.7 (0.3–1.5)	0.01	1.0 (0.4–2.2)	0.00	0.3 (0.1–0.9)	0.01	0.9 (0.4–2.0)	0.01	1.0 (0.6–1.7)	0.01	0.8 (0.4–1.6)
War, riots, killings (range: 0–3)	0.07	5.1 (3.4–7.5)	0.07	5.0 (3.5–7.1)	0.07	5.2 (3.0–8.9)	0.07	5.8 (3.9–8.6)	0.08	6.2 (5.0–7.8)	0.07	5.4 (4.1–6.9)
Forced to leave home or care setting (range: 0–2)	0.03	3.4 (1.9–6.0)	0.04	3.5 (2.1–5.8)	0.03	3.4 (1.6–7.0)	0.01	1.0 (0.6–1.9)	0.01	1.0 (0.6–1.8)	0.01	1.1 (0.6–1.9)
Violence in family or care setting (range: 0–3)	0.07	6.6 (4.8–9.0)	0.08	7.5 (4.9–11.4)	0.06	5.4 (4.0–7.3)	0.09	9.1 (6.6–12.3)	0.09	8.8 (7.1–10.8)	0.10	9.4 (7.7–11.4)
Physical or sexual abuse (range: 0–4)	0.15	12.9 (9.6–17.4)	0.16	13.6 (9.9–18.5)	0.14	12.0 (8.5–17.1)	0.24	19.4 (14.5–26.1)	0.25	19.8 (17.6–22.2)	0.23	19 (16.7–21.7)
Witnessing a family death (range: 0–2)	0.04	3.6 (2.5–5.2)	0.04	3.5 (2.3–5.2)	0.05	3.9 (2.5–6.2)	0.07	6.0 (4.1–8.6)	0.07	6.2 (5.0–7.8)	0.07	5.6 (4.5–7.0)
Any Trauma (range: 0–17)	0.38	23.6 (19.4–28.7)	0.40	23.6 (19.1–29.3)	0.35	23.6 (18.6–30.0)	0.50	30 (24.7–36.5)	0.51	30.7 (28.0–33.6)	0.48	29.3 (26.8–32.0)

a A category was considered endorsed if any of the items in it was endorsed. The total number of items for a category is indicated by the range.

b Mean number of items endorsed in the category.

**Fig. 1.**
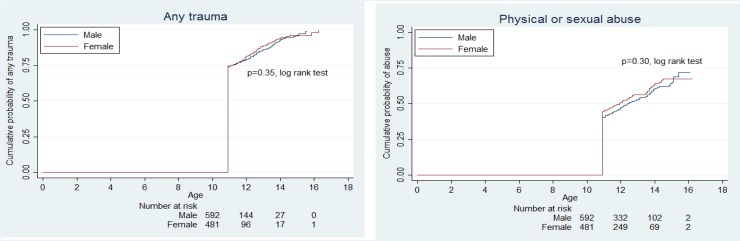
(*a, b*) Incidence of traumatic events in OSC ages ≥10.

Figure 1 shows Kaplan–Meier estimates of the failure (cumulative probability) functions of trauma and abuse by age for males and females. Approximately 75% of both male and female OSC had experienced at least one traumatic event by age 10, with a cumulative probability estimate of virtually 100% by age 15 (panel A). Approximately 45% of both male and female OSC had experienced physical or sexual abuse by age 10, with a cumulative probability estimate of approximately 70% by age 15 (panel B). There was little evidence of gender differences in the cumulative hazard of trauma over time (any trauma: *p* = 0.35; physical or sexual abuse: *p* = 0.30, log-rank tests).

### Anxiety outcome

**Fig. 2.**
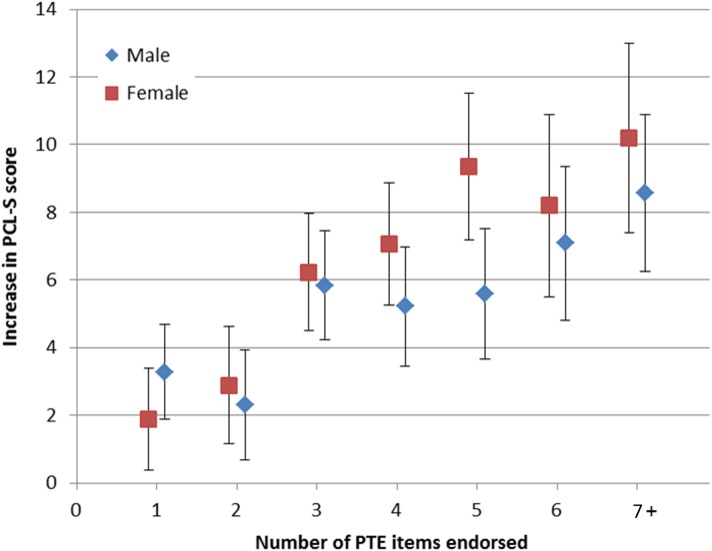
Change in anxiety score associated with increasing numbers of PTEs experienced.

Figure 2 shows the change in anxiety score associated with increasing numbers of PTEs experienced, ranging from 0 (referent) to 7 or more items endorsed. For both boys and girls, self-reported anxiety increased as the number of PTEs increased, although there were no statistically significant differences between boys and girls.

### Missing data analyses

Of the 2608 children who were theoretically eligible for inclusion in the analysis (were ≥10 years of age at some point during follow-up), 2235 out of 2608 (14% missing) contributed information on trauma prevalence and 2219 of 2608 (15% missing) contributed information on prevalence. The missingness on the number of eligible interviews was higher: 5052 of 7402 (32% missing) records had complete prevalence information and 4661 of 7401 (37% missing) had complete incidence information.

Using the inverse-probability-weighted data, estimates across settings and across genders were very similar to the observed data. Inverse probability weighted prevalence estimates differed by <3% and inverse probability weighted incidence estimates differed by <2% from the observed values. The relative comparisons of males and females across settings and the overall conclusions were the same, as those from the observed data.

## Discussion

Our study showed that OSC have a very high lifetime prevalence of PTEs. Furthermore, during longitudinal follow-up, nearly a quarter of institution-based and 30% of family-based OSC per year experienced an incident PTE. We found very similar lifetime prevalence and annual incidence of PTEs in general, and sexual and physical abuse in particular, among girls and boys.

By far, the trauma with the greatest annual incidence for both genders was physical and sexual abuse, affecting 12% of girls and 14% of boys in institution-based care and 19% of girls and 20% of boys in family-based care. Lifetime prevalence of such abuse was approximately 50% for both genders, regardless of setting. Yet, international funding mechanisms often place a special emphasis on the protection of girls while neglecting to discuss the special needs of boys for protection from physical and sexual abuse. For example, the US President's Emergency Plan for AIDS Relief, which is the largest single payer of care for orphans and vulnerable children (OVC), focuses on discussions of gender inequities and the need for special services for girls, but not protection for boys (United States Government, 2012). Similarly, the United Nations (UN) task force on protection from sexual exploitation and abuse focuses on the imperative to protect women and girls, but does not directly address needs of boys, although the UN elsewhere documents that 73 million boys along with 150 million girls have been sexually abused (United Nations, 2006, 2010). Our analyses support the need to protect both girls as well as boys.

The overall burden of PTEs experienced by OSC is substantial, and has implications for health, quality of life and economic productivity in adulthood. Associations between early traumatic events and adverse outcomes such as post-traumatic stress disorder, depression, re-victimization and decreased medication adherence have been well-documented (Koenig *et al.* 2004; Kounou *et al*. 2013; Whetten *et al*. 2013; Buhmann, 2014). Our analysis showing increase in anxiety associated with additional PTEs is consistent with existing studies.

Prior studies evaluating gender differences in the experience of PTEs among children have shown varying results. A meta-analysis of 40 studies indicated female gender as a predictor of post-traumatic stress in children, but noted a small effect size (Alisic *et al*. 2011). Studies that more closely reflect our population (OSC in LMIC) had results more consistent with the present study in observing comparable prevalence of PTEs across genders (Li *et al.* 2009; Atwoli *et al.* 2014).

Identification and support services for survivors of PTEs are essential to mitigate the long-term impact of these experiences. While a number of studies documenting such services in LMIC have been published, these studies generally had a primary focus on girls. For example, four studies examining medical care and counseling services related to assault in sub-Saharan Africa were either exclusively focused on women and girls, or reflected a patient population suggesting that most or all assault victims were female (Chaudhry *et al.* 1995; Ekabua *et al.* 2006; Kim *et al.* 2009; Ige & Fawole, 2012). Comparable studies with a focus on male survivors of PTEs in these settings were not identified. Similarly, prevention programs in LMIC aimed at reducing the incidence of traumatic events, in particular sexual assault, have also primarily targeted girls (Sarnquist *et al.* 2014; Sommarin *et al.* 2014); again, comparable programs for boys were not identified. Our results suggest that while support services and prevention programs for girls are essential, similar programs for boys are likely equally important and may represent an important current gap.

Stigma surrounding abuse may be particularly acute in boys, preventing them from seeking services, and by extension, preventing them from being captured in studies of survivor services. A meta-analysis of adult male assault victims in the USA concluded that male sexual assault victims had greater stigma and fewer service options than female counterparts; a separate systematic review also concluded sexual assault in boys was common but underreported, under-recognized and undertreated (Holmes & Slap, 1998; Bullock & Beckson, 2011). One qualitative study in the Democratic Republic of Congo similarly documented experiences of male survivors of sexual and gender-based violence related to armed conflicts. The authors observed stigma and under-reporting by male victims, as well as a lack of available services relative to female counterparts (Christian *et al.* 2011). Additional research is needed on whether OSC boys in LMIC experience additional stigma associated with abuse, whether and how they access survivor services, and what prevention and intervention programs may be most useful to them.

Our study is strengthened by several key features. The study population was drawn from six sites in five culturally, politically and geographically diverse LMIC. We used longitudinal data from a statistically representative cohort of OSC living in both family-based and institution-based settings. The prospective design enabled estimation of both lifetime prevalence and incidence of traumatic events. Finally, the POFO study had a high percentage of 3-year follow-up (80.5%), and had complete data on non-trauma-related variables (gender, setting, study site and age).

There are also several limitations to consider. First, traumatic experiences are likely to be under-reported, meaning our estimates may be lower than the actual prevalence and incidence of PTEs in this population. Second, the frequency of events was reported as ‘none’, ‘one’ and ‘two or more’. Our analyses are limited in accounting for the complete burden of repeated events, which may vary by gender, though we have included analyses on ‘two or more’ events. Third, though our population is from diverse LMIC, South America and Eastern Europe are not represented, which limits generalizability to those settings. Fourth, for both prevalence and incidence analyses, approximately 85% of eligible children contributed at least one trauma interview. However, we had high missingness on the number of eligible trauma interviews (>30% for both prevalence and incidence). We assessed the impact of potential selection bias through inverse probability weighting. Our analyses indicated that missingness had minimal impact on the results: estimates differed from observed values by <3% in prevalence estimates and <2% in incidence estimates. Importantly, the overall conclusions were the same as the observed data: we observed no differences across gender or setting. Finally, assessing PTEs is challenging, particularly in different cultural settings and in children. We used items from the LEC because they have been shown to be predictive of PTSD, depression and anxiety and are widely used across cultures (Elhai *et al*. 2005). We limited our study population to OSC ≥10 to ensure self-reported answers were as accurate as possible; prior studies and our own pilot study indicated that younger children may not understand or be able to accurately report events. The Cronbach's alphas for the subcategories of potential traumatic events were low (0.23–0.56), as would be expected for combinations of small numbers of items, especially since experience of one type of event in a given subcategory would not necessarily be expected to correlate with experience of another type (e.g. exposure to hurricane and to motor vehicle accident). This suggests that the binary ‘any event experienced’ measure of each subcategory may be more appropriate than the count measure of number of types of events. The Cronbach's alpha for the continuous measure of all types of trauma experiences was higher (0.65).

As the number of OSC worldwide continues to grow, addressing their needs is an increasingly complex challenge. We have shown that the burden of PTEs is quite high in general, demanding the attention of policymakers developing interventions for this vulnerable population. Importantly, we have also shown that such interventions targeting OSC should not be limited to girls, but must include boys as well. Limiting interventions surrounding traumatic events to girls misses half of the population at risk.

## Appendix

**Table A1.** Specific items included in each trauma category

Category	Items in category
Disaster or accidents	1. Been in a big earthquake that damaged the building I was in
	2. Been in another disaster, like a fire, tornado, flood, hurricane, tsunami, big storm
	3. Been in a bad accident, like a car accident
War, riots or killings	1. Been in a place where war, conflict, fighting or riots were going on
	2. Saw someone in the town shot at or killed
	3. Seen someone hurt or killed during a war
Physical or sexual abuse	1. Been hit, kicked or beaten at home
	2. Been hit, kicked or beaten by other children
	3. Someone touched my private sexual parts when I did not want them to
	4. I was raped or sexually molested
Violence in family or care setting	1. Saw a family member hit, kicked or beaten
	2. Saw a family member shot at or killed
	3. I saw a family member raped or sexually molested
Witnessing a family death	1. Saw a family member die a violent death or be seriously injured
	2. Seen a family member die from illness
Forced to leave home or care setting	1. Was forced to move or run from home due to a war, conflict or fighting
	2. Was forced to move from home because there was no one (adult) there to stay with

## References

[ref1] Alisic E, Jongmans MJ, van Wesel F, Kleber RJ (2011). Building child trauma theory from longitudinal studies: a meta-analysis. Clinical Psychology Review 31, 736–47. 21501581 10.1016/j.cpr.2011.03.001

[ref2] American Psychiatric Association (2013). Diagnostic and Statistical Manual of Mental Disorders 5th ed. Washington, DC.

[ref3] Atwoli L, Ayuku D, Hogan J, Koech J, Vreeman RC, Ayaya S, Braitstein P (2014). Impact of domestic care environment on trauma and posttraumatic stress disorder among orphans in western Kenya. PLoS ONE 9, e3. 10.1371/journal.pone.0089937 PMC3953071 24625395

[ref4] Birdthistle IJ, Floyd S, Mwanasa S, Nyagadza A, Gwiza E, Glynn JR (2011). Child sexual abuse and links to HIV and orphanhood in urban Zimbabwe. Journal of Epidemiology and Community Health 65, 1075–82. 20628080 10.1136/jech.2009.094359

[ref5] Blanchard EB, Jones-Alexander J, Buckley TC, Forneris CA (1996). Psychometric properties of the PTSD Checklist (PCL). Behavior Research Therapy 34, 669–73. 10.1016/0005-7967(96)00033-2 8870294

[ref6] Buhmann CB (2014). Traumatized refugees: morbidity, treatment and predictors of outcome. Danish Medical Journal 61, B4871. 25162447

[ref7] Bullock CM, Beckson M (2011). Male victims of sexual assault: phenomenology, psychology, physiology. Journal of the American Academy of Psychiatry and the Law 39, 197–205. 21653264

[ref8] Chaudhry S, Sangani B, Ojwang SB, Khan KS (1995). Retrospective study of alleged sexual assault at the Aga Khan Hospital, Nairobi. East African Medical Journal 72, 200–2. 7796777

[ref9] Christian M, Safari O, Ramazani P, Burnham G, Glass N (2011). Sexual and gender based violence against men in the Democratic Republic of Congo: effects on survivors, their families and the community. Medicine, Conflict and Survival 27, 227–46. 10.1080/13623699.2011.645144 22416570

[ref10] Ekabua JE, Agan TU, Iklaki CU, Ekanem EI, Itam IH, Ogaji DS (2006). Trauma related to sexual assault in Calabar, south eastern Nigeria. Nigerian Journal of Medical 15, 72–4. 10.4314/njm.v15i1.37121 16649457

[ref11] Escueta M, Whetten K, Ostermann J, O'donnell K (2014). Adverse childhood experiences, psychosocial well-being and cognitive development among orphans and abandoned children in five low income countries. BMC International Health & Human Rights 14, 6. 24606949 10.1186/1472-698X-14-6 PMC3975306

[ref12] Elhai JD, Gray MJ, Kashdan TB, Franklin CL (2005). Which instruments are most commonly used to assess traumatic event exposure and posttraumatic effects?: A survey of traumatic stress professionals. Journal of Traumatic Stress 18, 541–545. 16281252 10.1002/jts.20062

[ref13] Gray MJ, Litz BT, Hsu JL, Lombardo TW (2004). Psychometric properties of the life events checklist. Assessment 11, 330–41. 15486169 10.1177/1073191104269954

[ref14] Holmes WC, Slap GB (1998). Sexual abuse of boys: definition, prevalence, correlates, sequelae, and management. Journal of the American Medical Association 280, 1855–62. 9846781 10.1001/jama.280.21.1855

[ref15] Ige O, Fawole OI (2012). Evaluating the medical care of child sexual abuse victims in a general hospital in Ibadan, Nigeria. Ghana Medical Journal 46, 22–6. 22605885 PMC3353498

[ref16] Kim JC, Askew I, Muvhango L, Dwane N, Abramsky T, Jan S, Ntlemo E, Chege J, Watts C (2009). Comprehensive care and HIV prophylaxis after sexual assault in rural South Africa: the Refentse intervention study. British Medical Journal 338, b515. 19286746 10.1136/bmj.b515

[ref17] Koenig L, Doll L, O'leary A, Pequegnat W (eds.) (2004). From Child Sexual Abuse to Adult Sexual Risk: Trauma, Revictimization, and Intervention. American Psychological Association: Washington, DC.

[ref18] Kounou KB, Bui E, Dassa KS, Hinton D, Fischer L, Djassoa G, Birmes P, Schmitt L (2013). Childhood trauma, personality disorders symptoms and current major depressive disorder in Togo. Social Psychiatry Psychiatric Epidemiology 48, 1095–103. 23224674 10.1007/s00127-012-0634-2

[ref19] Li X, Barnett D, Fang X, Lin X, Zhao G, Zhao J, Hong Y, Zhang L, Naar-King S, Stanton B (2009). Lifetime incidences of traumatic events and mental health among children affected by HIV/AIDS in rural China. Journal of Clinical Child & Adolescent Psychology 38, 731–44. 20183657 10.1080/15374410903103601

[ref20] Mugavero M, Ostermann J, Whetten K, Leserman J, Swartz M, Stangl D, Thielman N (2006). Barriers to antiretroviral adherence: the importance of depression, abuse, and other traumatic events. AIDS Patient Care STDS 20, 418–28. 16789855 10.1089/apc.2006.20.418

[ref21] Rajan DG, Shirey K, Ostermann J, Whetten R, O'Donnell K, Whetten K (2013). Child and caregiver concordance of potentially traumatic events experienced by orphaned and abandoned children. Vulnerable Children and Youth Studies: An International Interdisciplinary Journal for Research, Vulnerable Children and Youth Studies 9(3), 220–233. doi: 10.1080/17450128.2013.855346. PMC4217223 25379051

[ref22] Sarnquist C, Omondi B, Sinclair J, Gitau C, Paiva L, Mulinge M, Cornfield DN, Maldonado Y (2014). Rape prevention through empowerment of adolescent girls. *Pediatrics* 133(5), e1226–32. 10.1542/peds.2013-3414 24733880

[ref23] Seaman SR, White IR (2013). Review of inverse probability weighting for dealing with missing data. Statistical Methods in Medical Research 22, 277–95. 10.1177/0962280210395740 21220355

[ref24] Sommarin C, Kilbane T, Mercy JA, Moloney-Kitts M, Ligiero DP (2014). Preventing sexual violence and HIV in children. Journal of Acquired Immune Deficiency Syndromes 66(Suppl 2), S217–23. 24918598 10.1097/QAI.0000000000000183 PMC5889850

[ref25] StataCorp. (2013). Stata Statistical Software: Release 13. StataCorp LP: College Station, TX.

[ref26] United Nations (2006). Protection from sexual exploitation and abuse. http://www.unviolencestudy.org/ Accessed November 25, 2014.

[ref27] United Nations (2010). Protection from sexual exploitation and abuse. http://www.un.org/en/pseataskforce/overview.shtml Accessed November 25, 2014.

[ref28] United States Government (2012). President's emergency plan for AIDS relief. http://www.pepfar.gov/documents/organization/195702.pdf Accessed November 25, 2014.

[ref29] UNICEF (2012). *State of the World's Children* http://www.unicef.org/sowc2012/pdfs/SOWC%202012-Main%20Report_EN_13Mar2012.pdf Accessed April 25, 2014.

[ref30] Weathers FW, Litz BT, Herman DS, Huska JA, Keane TM (1991). The PTSD Checklist-Civilian Version (PCLC). National Center for PTSD, Boston Veteran's Affairs Medical Center: Boston, MA.

[ref31] Weathers FW, Litz BT, Herman DS, Huska JA, Keane TM (1993). *Reliability, validity, and diagnostic utility.* Paper presented at the Trauma, Coping and Adaptation ISTSS: 9^th^ Annual Meeting, San Antonio, TX.

[ref32] Whetten K, Ostermann J, Whetten R, O'donnell K, Thielman N (2011). More than the loss of a parent: potentially traumatic events among orphaned and abandoned children. Journal of Trauma Stress 24, 174–82. 10.1002/jts.20625 PMC3610328 21442663

[ref33] Whetten K, Ostermann J, Whetten RA, Pence BW, O'donnell K, Messer LC, Thielman NM (2009). A comparison of the wellbeing of orphans and abandoned children ages 6–12 in institutional and community-based care settings in 5 less wealthy nations. PLoS ONE 4, e3. 10.1371/journal.pone.0008169 PMC2790618 20020037

[ref34] Whetten K, Shirey K, Pence BW, Yao J, Thielman N, Whetten R, Adams J, Agala B, Ostermann J, O'donnell K, Hobbie A, Maro V, Itemba D, Reddy E (2013). Trauma history and depression predict incomplete adherence to antiretroviral therapies in a low income country. PLoS ONE 8, e3. 10.1371/journal.pone.0074771 PMC3790775 24124455

